# Incremental Value of Left Ventricular Mechanical Dyssynchrony Assessment by Nitrogen-13 Ammonia ECG-Gated PET in Patients With Coronary Artery Disease

**DOI:** 10.3389/fcvm.2021.719565

**Published:** 2021-10-15

**Authors:** Danzha Zheng, Yanyun Liu, Lei Zhang, Fan Hu, Xubo Tan, Dawei Jiang, Weihua Zhou, Xiaoli Lan, Chunxia Qin

**Affiliations:** ^1^Department of Nuclear Medicine, Union Hospital, Tongji Medical College, Huazhong University of Science and Technology, Wuhan, China; ^2^Hubei Key Laboratory of Molecular Imaging, Wuhan, China; ^3^Engineering Research Center of Molecular and Neuro Imaging of Ministry of Education, School of Life Science and Technology, Xidian University, Xi'an, China; ^4^Department of Applied Computing, Michigan Technological University, Houghton, MI, United States; ^5^Center of Biocomputing and Digital Health, Institute of Computing and Cybersystems, and Health Research Institute, Michigan Technological University, Houghton, MI, United States

**Keywords:** positron emission tomography, myocardial perfusion imaging (MPI), left ventricular mechanical dyssynchrony, coronary artery disease, myocardial viability

## Abstract

**Background:** Phase analysis is a technique used to assess left ventricular mechanical dyssynchrony (LVMD) in nuclear myocardial imaging. Previous studies have found an association between LVMD and myocardial ischemia. We aim to assess the potential diagnostic value of LVMD in terms of myocardial viability, and ability to predict major adverse cardiac events (MACE), using Nitrogen-13 ammonia ECG-gated positron emission tomography (gPET).

**Methods:** Patients with coronary artery disease (CAD) who underwent Nitrogen-13 ammonia and Fluorine-18 FDG myocardial gPET were enrolled, and their gPET imaging data were retrospectively analyzed. Patients were followed up and major adverse cardiac events (MACE) were recorded. The Kruskal-Wallis test and Mann-Whitney U test were performed to compare LVMD parameters among the groups. Binary logistic regression analysis, receiver operating characteristic (ROC) curve analysis, and multiple stepwise analysis curves were applied to identify the relationship between LVMD parameters and myocardial viability. Kaplan–Meier survival curves and the log-rank test were used to look for differences in the incidence of MACE.

**Results:** In total, 79 patients were enrolled and divided into three groups: Group 1 (patients with only viable myocardium, *n* = 7), Group 2 (patients with more viable myocardium than scar, *n* = 33), and Group 3 (patients with less viable myocardium than scar, *n* = 39). All LVMD parameters were significantly different among groups. The median values of systolic phase standard deviation (PSD), systolic phase histogram bandwidth (PHB), diastolic PSD, and diastolic PHB between Group 1 and Group 3, and Group 2 and Group 3 were significantly different. A diastolic PHB of 204.5° was the best cut-off value to predict the presence of myocardial scar. In multiple stepwise analysis models, diastolic PSD, ischemic extent, and New York Heart Association (NYHA) classification were independent predictive factors of viable myocardium and myocardial scar. The incidence of MACE in patients with diastolic PHB > 204.5° was 25.0%, higher than patients with diastolic PHB <204.5° (11.8%), but the difference was not significant.

**Conclusions:** LVMD generated from Nitrogen-13 ammonia ECG-gated myocardial perfusion imaging had added diagnostic value for myocardial viability assessment in CAD patients. LVMD did not show a definite prognostic value.

## Introduction

Evaluation of myocardial viability is important in patients with coronary artery disease (CAD) when planning revascularization (1) and predicting the improvement of LV systolic function (2). The imaging methods to assess myocardial viability include coronary angiography, echocardiography, cardiac computed tomography (CT), cardiovascular magnetic resonance imaging, and nuclear myocardial imaging (1). Myocardial viability evaluated by myocardial perfusion imaging (MPI) and glucose metabolism imaging is regarded as the “gold standard” of non-invasive imaging (3). Compared with conventional imaging methods (4), myocardial perfusion and metabolism imaging assess regional myocardial physiology and function directly. However, the cost of both myocardial perfusion and metabolism positron emission tomography (PET) imaging is very high. The process to regulate blood glucose levels is complicated, especially in patients with diabetes (4). After time-consuming preparation, the image quality of some patients is still not ideal. Therefore, it is necessary to look for an alternative method to distinguish viable myocardium from myocardial scar.

Phase analysis has emerged as an important technique to assess left ventricular mechanical dyssynchrony (LVMD) using nuclear imaging. Studies of the application of phase analysis have concentrated on the use of single-photon emission computed tomography (SPECT), in areas such as LVMD assessment in patients with left bundle branch block (5), optimizing patient selection for cardiac resynchronization therapy (CRT) (6), and improving CRT response (7, 8). Several studies have investigated the application of phase analysis for early diagnosis (9, 10), therapy (11), and prognosis evaluation (12, 13) in CAD patients. The worse degree of LVMD under stress perfusion imaging compared to images at rest was positively correlated with ischemic extent (14). Hibernating myocardium was an independent predictive factor of LVMD (15). So LVMD may have some relationship with ischemic myocardium and myocardial viability.

Limited studies have focused on gPET (16–18), especially using Nitrogen-13 ammonia ECG-gated MPI. PET can provide high-quality images with higher count rates, and increased spatial resolution compared with SPECT (4), and more positron tracers with ideal performance are available (19). The purpose of this study was to identify the diagnostic value of LVMD assessed with gPET MPI for myocardial viability assessment and its prognostic value in CAD patients.

## Materials and Methods

### Study Population

*This study was approved by the Institutional Ethical Committee of the Union Hospital, Tongji Medical College, Huazhong University of Science and Technology*. Patients who underwent Nitrogen-13 ammonia gPET and Fluorine-18 FDG gPET for the myocardial viability assessment at the PET Center of Union Hospital from December 2015 to October 2019 were retrospectively enrolled. The inclusion criteria were adult patients with CAD as the main diagnosis confirmed by coronary angiography or coronary CT angiography. The exclusion criteria were: (1) severe valvular heart disease, (2) non-ischemic cardiomyopathy, and (3) severe arrhythmias such as left bundle branch block. Age, sex, history of myocardial infarction (MI) or old MI, risk factors for CAD, New York Heart Association (NYHA) functional classification were recorded. All the patients were followed up via medical records or telephone review to record the incidence of major adverse cardiac events (MACE), including unstable angina pectoris, myocardial infarction, heart failure, PCI, CABG, stroke, and cardiac death.

### Imaging Acquisition

All patients underwent a rest Nitrogen-13 ammonia gPET and Fluorine-18 FDG gPET with a PET/CT scanner (GE Discovery VCT^®^, GE Healthcare, Milwaukee WI, USA) using a 1-day protocol. Aminophylline and caffeinated beverages were avoided for 48 h before the PET examination. All patients fasted for 6 h. After connection of electrocardiographic (ECG) gating, and immediately after intravenous injection of 370–740 MBq (10-20 mCi) Nitrogen-13 ammonia, rest Nitrogen-13 ammonia static PET images were acquired for 10 min. Subsequently, oral glucose-loading with 25–50 g was performed, with supplemental insulin administered as needed (4). Between 1 h and 90 min after intravenous injection of 185–370 MBq (5–10 mCi) Fluorine-18 FDG, PET data were acquired for 10 min under ECG gating. Images were generated using volume image protocol (VIP) replay with eight frames per R-to-R interval (4), screening out the frames with heart rates within the upper and lower 20%. The images were reconstructed using the ordered subsets expectation maximization (OSEM) method.

### Imaging Analysis

The data were input into commercial software (Emory Cardiac Toolbox®, ECTb, Atlanta GA, USA) (20) to measure left ventricular (LV) functional parameters, LV wall thickening score, LVMD parameters, ischemic extent, and myocardial viability, including the degree of viable myocardium and myocardial scar.

#### Myocardial Viability Assessment and Grouping

The ischemic extent was assessed on Nitrogen-13 ammonia MPI, obtained from ECTb software by using the default threshold of 50% (20). The algorithm of ECTb software searches for the maximal counts in the entire LV myocardial distribution and identifies those myocardial segments that fall below 50% of this maximal value as ischemic, using the extensively validated method of O'Connor et al. (21). Ischemic extent was defined as the ratio of ischemia area to the whole LV area. The areas with normal FDG uptake were defined by comparing with the normal database provided by software developer, and the FDG uptake was scaled to equate its average value in the normal region with that in the perfusion study. The FDG tool differentiate viable myocardium and myocardial scar by a threshold of 40% of the maximum FDG counts in LV myocardium. In the area with FDG distribution above the threshold coexistent with relatively decreased perfusion was considered viable myocardium (perfusion/metabolic mismatch), under the threshold coexistent with decreased perfusion was considered as myocardial scar (perfusion/metabolic match) (4, 20). The degree of viable myocardium/myocardial scar was defined as the ratio of viable myocardium area/scar area to the whole LV area. CAD Patients were grouped by comparison of their own degree of viable myocardium and myocardial scar. Group 1 included patients with viable myocardium only. Group 2 included patients with more viable myocardium than their own myocardial scar. Group 3 included patients with more myocardial scar than their own viable myocardium.

#### LVMD Analysis

The software obtained the change of myocardial wall thickness by the change in maximum counts at the same myocardial region. After matching the onset time when the myocardial region starts contracting, the time distribution of myocardial contraction percentage in the cardiac cycle was obtained (22). The phase histogram was used to display the correlation of phase and the percentage of myocardial contraction, with the *x*-axis representing the phase (time), and the *y*-axis representing the percentage of myocardium that starts contracting at the corresponding time on the *x*-axis. Phase standard deviation (PSD) and phase histogram bandwidth (PHB) were the most common parameters to describe systolic LVMD, as the standard deviation and 95% width of phase distribution, respectively (22). Similar methods were used to assess LVMD at LV diastole. The LV wall thickening curve was approximated by the 3-harmonic function for diastolic LVMD after undergoing a count drop correction. Use diastolic PSD and PHB to describe diastolic LVMD (23).

### Statistical Analysis

Data were processed by commercial statistical software (IBM SPSS Statistics 25.0, IBM, Armonk NY, USA). Normally distributed continuous variables are expressed as mean ± standard deviation, non-normally distributed continuous variables as median and interquartile range (25th, 75th), and categorical variables as numbers or percentages. Either analysis of variance or the Kruskal–Wallis test was used to compare LV functional and LVMD parameters among groups. Mann-Whitney U test was performed for pairwise comparisons. Binary logistic and multiple stepwise regression analyses were performed to screen for parameters predictive of myocardial viability. A receiver operating characteristic (ROC) curve was generated. The cut-off value was determined by the Youden index. Kaplan–Meier survival curve and log-rank test were generated to assess for differences in the incidence of MACE. *P* < 0.05 was considered statistically significant.

## Results

### Patient Characteristics

**Figure 1:**
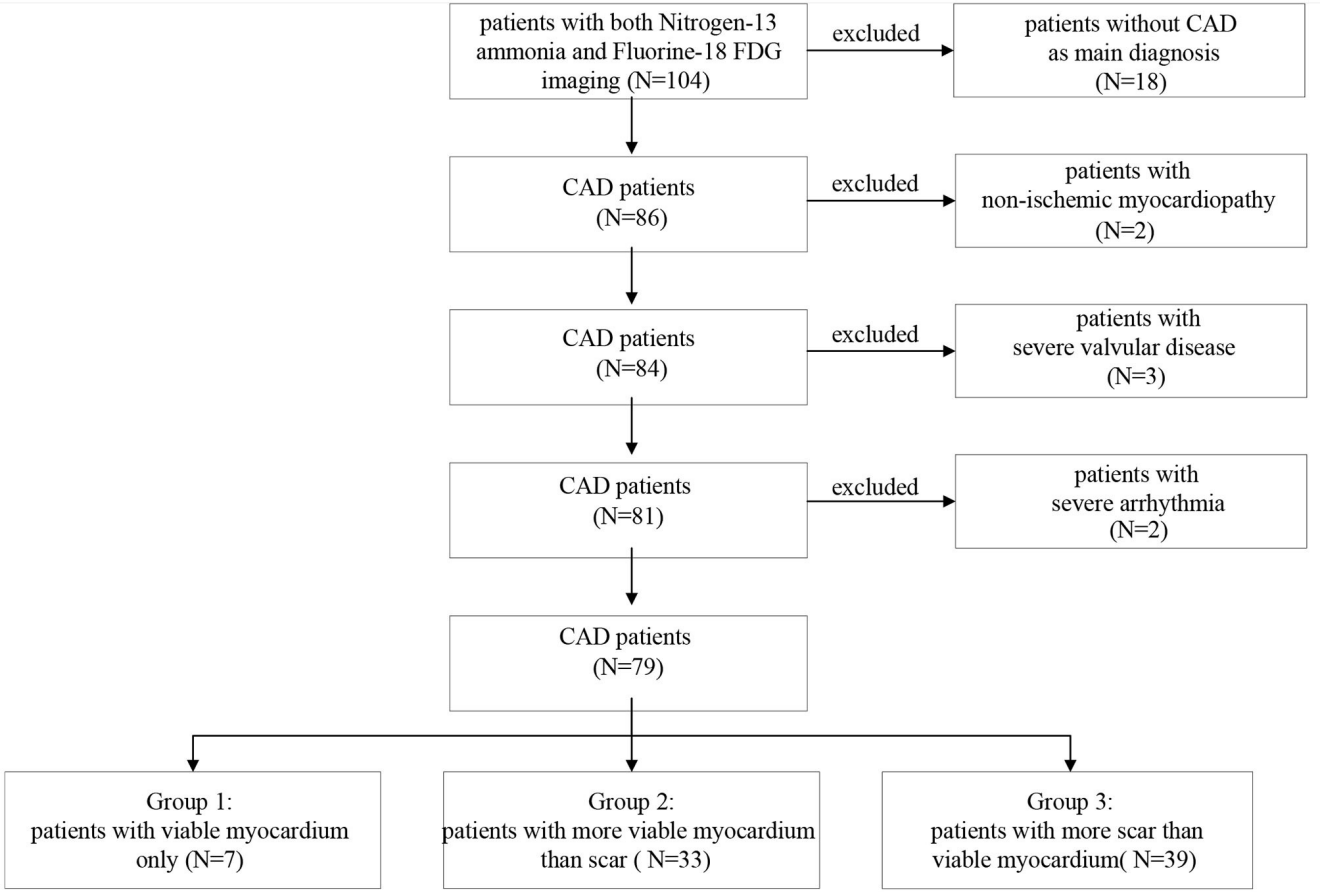
Screening and grouping process of patients with coronary artery disease (CAD).

A total of 104 patients underwent both Nitrogen-13 ammonia and Fluorine-18 FDG myocardial gPET imaging; 86 patients were diagnosed with CAD. After excluding two cases of non-ischemic cardiomyopathy, three cases of severe valvular disease, and two cases with severe arrhythmia, a total of 79 patients were included in this study (Figure 1). Age, sex, MI or old MI, the proportion of overweight and obese, risk factors for CAD, LV functional parameters, LVMD parameters, wall thickening scores, and myocardial viability assessments of all patients are shown in Table 1. NYHA functional classification III and IV were the most common functional classification in patients. In addition, LV systolic dysfunction and regional wall motion abnormality commonly existed in the study population.

**Table 1:** Basic characteristics of the CAD patients [proportion, mean ± SD or median (25th, 75th)].

	**CAD patients (*N* = 79)**
Age (y)	56.8 ± 9.9
Male	68/79 (86.1%)
Overweight (25 < = BMI <30)	27/79 (34.2%)
Obese (BMI > 30)	6/79 (7.6%)
MI or Old MI	45/79 (57.0%)
**CARDIAC RISK FACTORS OF CAD**	
Family history of cardiovascular disease	15/79 (19.0%)
Smoke	45/79 (57.0%)
Hyperlipemia	19/79 (24.1%)
Hypertension	40/79 (50.6%)
Diabetes	31/79 (39.2%)
**NYHA FUNCTIONAL CLASSIFICATION**	
II	15/79 (19.0%)
III	41/79 (51.9%)
IV	23/79 (29.1%)
**LEFT VENTRICULAR FUNCTIONAL PARAMETERS**	
EF (%)	26.0 (19.0, 36.0)
EDV (ml)	242.0 (180.0, 334.0)
ESV (ml)	178.0 (118.0, 251.0)
PFR (EDV/RR)	1.2 (0.9, 1.6)
TPFR (%RR)	23.2 (15.1, 38.2)
**REGIONAL WALL MOTION PARAMETERS**	
Systolic PSD (°)	77.1 (62.2, 85.2)
Systolic PHB (°)	252.0 (201.0, 283.0)
Diastolic PSD (°)	81.3 (66.9, 90.0)
Diastolic PHB (°)	268.0 (213.0, 295.0)
Wall thickening score (%)	11.3 ± 7.3
**MYOCARDIAL VIABILITY PARAMETERS**	
Ischemic extent (%)	25.8 ± 11.5
Viable myocardium (%)	11.2 (5.4, 20.4)
Myocardial scar (%)	7.6 (2.3, 22.8)

*CAD, coronary artery disease; SD, standard deviation; PSD, phase standard deviation; PHB, phase histogram bandwidth; EF, ejection fraction; EDV, end-diastolic volume; ESV, end-systolic volume; PFR, peak filling rate; TPFR, time of peak filling rate; RR, RR interval*.

### Comparison of LV Functional and LVMD Parameters Among Groups

There were 7 patients in Group 1, 33 in Group 2, and 39 in Group 3 (Figure 1). The number of patients with MI was 6 (85.7%), 14 (42.4%), and 25 (64.1%) in each group, respectively. Among them, 2 (28.6%) in Group 1, 9 (27.3%) in Group 2 and 19 (48.7%) in Group 3 had old MI. In the comparison of LV functional parameters, there were no significant differences among the three groups in LV ejection fraction (LVEF), end-diastolic volume (EDV), end-systolic volume (ESV), peak filling rate (PFR), or time of peak filling rate (TPFR) (Table 2).

**Table 2:** Comparison of left ventricular functional parameters among groups [median (25th,75th)].

	**Group 1**	**Group 2**	**Group 3**	**H**	***P*-value**
EF (%)	29.0 (23.0, 65.0)	26.0 (19.0, 37.0)	24.0 (19.0, 34.0)	3.047	0.218
EDV (ml)	207.0 (87.0, 341.0)	242.0 (162.0, 363.0)	247.0 (196.0, 283.0)	1.808	0.405
ESV (ml)	146.0 (29.0, 262.0)	187.0 (114.5, 289.0)	193.0 (141.0, 229.0)	2.001	0.368
PFR (EDV/RR)	1.9 (1.1, 2.7)	1.1 (0.9, 1.4)	1.2 (0.9, 1.6)	2.404	0.301
TPFR (%RR)	22.5 (16.6, 28.7)	24.8 (15.3, 40.5)	21.3 (14.5, 38.1)	0.349	0.840

*Group 1, patients with viable myocardium only; Group 2, patients with more viable myocardium than scar; Group 3, patients with more scar than viable myocardium. EF, ejection fraction; EDV, end-diastolic volume; ESV, end-systolic volume; PFR, peak filling rate; TPFR, time of peak filling rate*.

**Figure 2:**
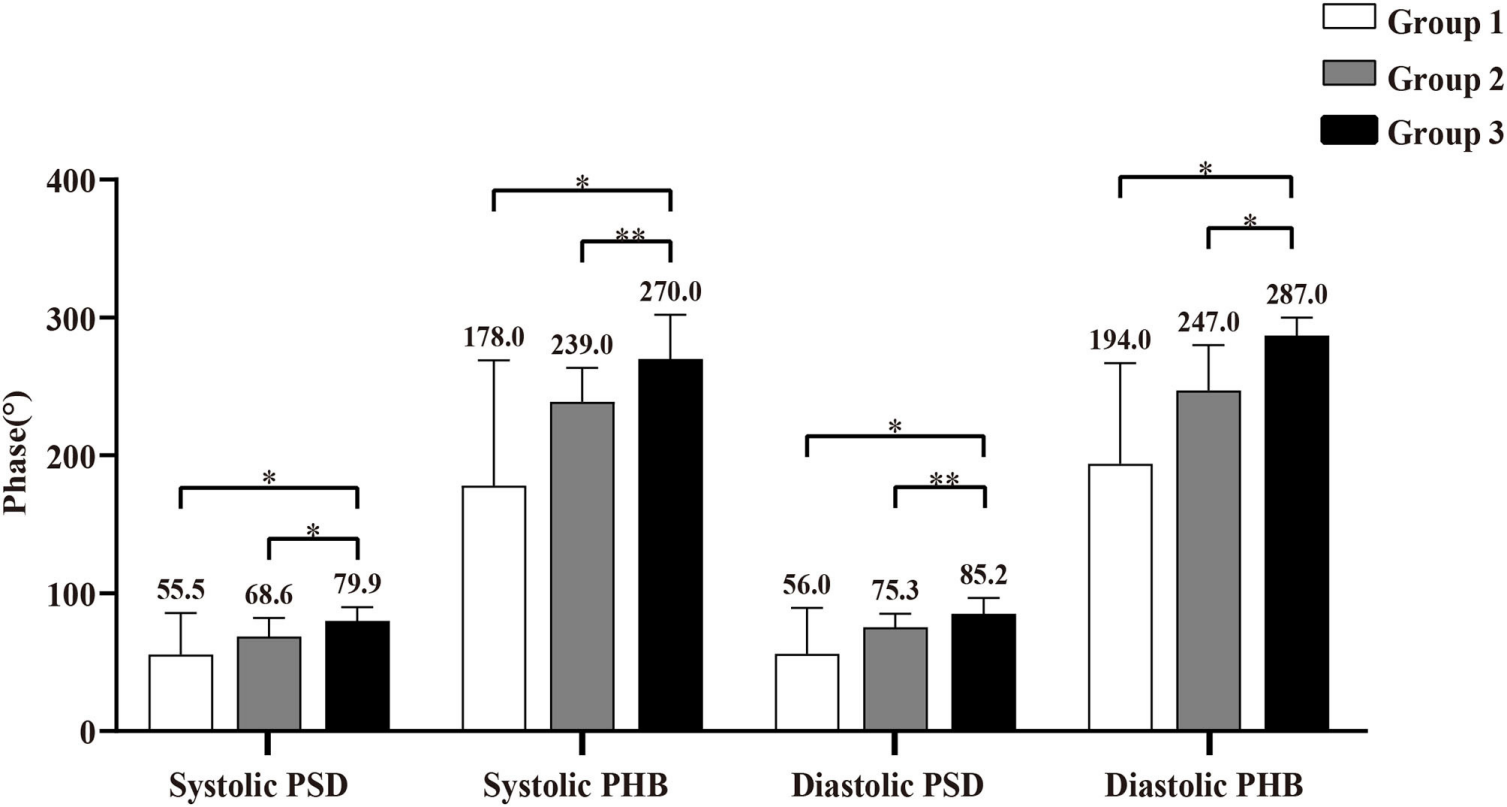
Comparison of left ventricular mechanical dyssynchrony (LVMD) parameters in Group 1 (patients with viable myocardium only), Group 2 (patients with more viable myocardium than scar), and Group 3 (patients with more scar than viable myocardium). * Indicates *P* < 0.05, and ** indicates *P* < 0.01. PSD, phase standard deviation; PHB, phase histogram bandwidth.

**Figure 3:**
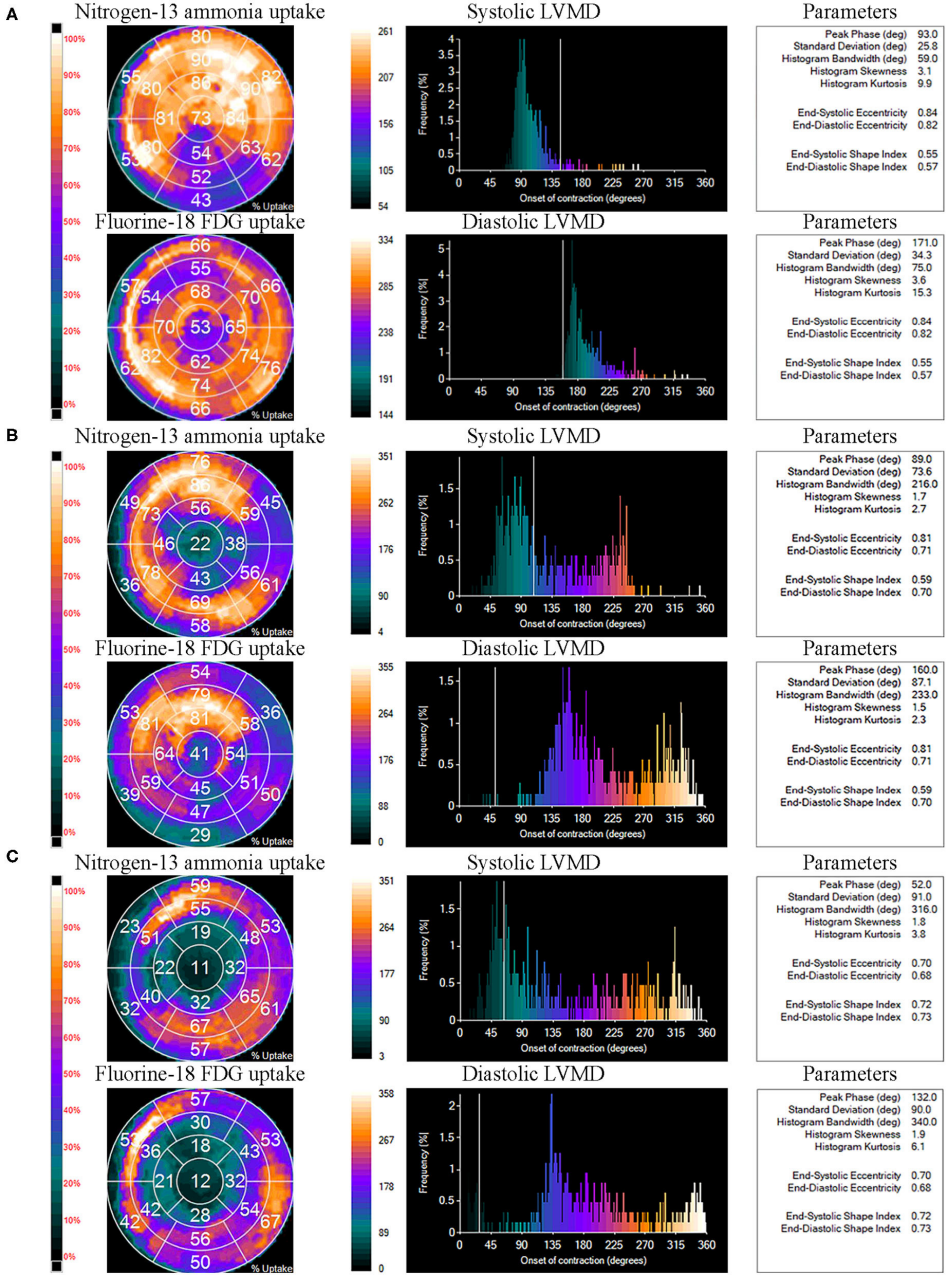
Examples of left ventricular mechanical dyssynchrony (LVMD) analysis of Group 1 **(A)**, Group 2 **(B)**, and Group 3 **(C)**. The panels tagged “Nitrogen-13 ammonia uptake” and “Fluorine-18 FDG uptake” represent the percentage of Nitrogen-13 ammonia and Fluorine-18 FDG uptake by each myocardial segment on LV polar maps. The panels tagged “systolic LVMD” and “diastolic LVMD” are the phase histograms of systolic LVMD analysis and diastolic LVMD analysis, respectively. Relative LVMD parameters were also output on the right.

The comparisons of LVMD parameters, including systolic PSD, systolic PHB, diastolic PSD, and diastolic PHB among groups were shown in Figure 2. All the LVMD parameters were statistically different among groups, the *P*-value of systolic PSD, systolic PHB, diastolic PSD, and diastolic PHB was 0.007, 0.001, 0.001, and 0.001, respectively. In pairwise comparison after Bonferroni's correction, the median values were significantly different between Group 1 and Group 3, *P* = 0.044, 0.017, 0.019, and 0.012 for systolic PSD, systolic PHB, diastolic PSD, and diastolic PHB, respectively. They were also significantly different between Group 2 and Group 3 (*P* = 0.030, 0.007, 0.005, and 0.010, respectively). No significant difference was found between Group 1 and Group 2. Examples of LVMD analysis were shown in Figure 3.

### Binary Logistic Regression Analysis and ROC Curves for Myocardial Viability

**Figure 4:**
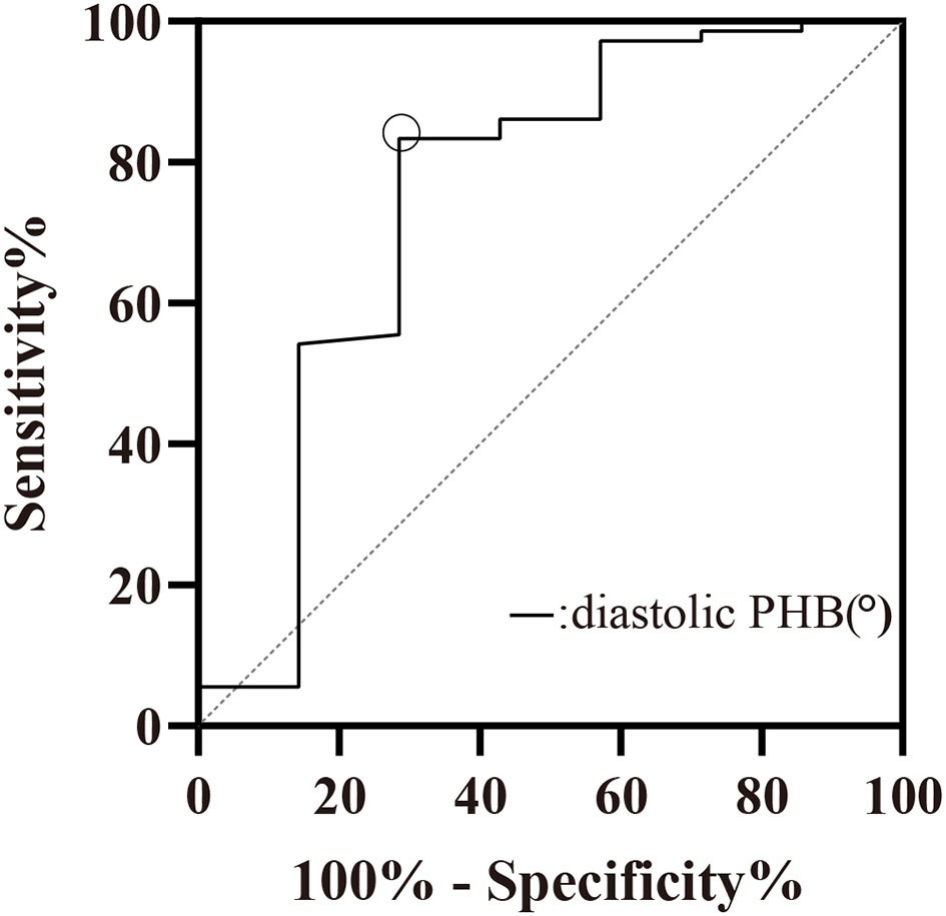
ROC curve of diastolic PHB (°) for the prediction of myocardial scar. Cut-off value: diastolic PHB > 204.5°, Sensitivity = 83.33%, Specificity = 71.43%. PHB, phase histogram bandwidth.

Age, sex, BMI, cardiac risk factors, NYHA functional classification, LV functional parameters, ischemic extent, wall thickening score, and LVMD parameters were included in the binary logistic regression model for predicting the presence of myocardial scar. Diastolic PHB was the only factor to identify myocardial scar, Odds ratio (95% Confidence Interval) = 1.015 (1.005–1.025), *P* = 0.004. The cut-off value for diastolic PHB to discriminate between patients with and without myocardial scar was 204.5°, the Youden index = 0.548 (Figure 4). The sensitivity and specificity were 83.3 and 71.4%, respectively. The area under the ROC curve was 0.751, *P* = 0.029.

### Multiple Stepwise Regression Analysis for the Prediction of the Myocardial Viability

Age, sex, BMI, cardiac risk factors, NYHA functional classification, LV functional parameters, ischemic extent, wall thickening score, and LVMD parameters were included in the multiple regression stepwise model to predict viable myocardium and myocardial scar. Ischemic extent, diastolic PSD, and NYHA functional classification were identified as the independent factors to predict viable myocardium (coefficient of determination [*R*^2^] = 0.329) and myocardial scar (*R*^2^ = 0.373), as shown in Table 3.

**Table 3:** The multiple stepwise regression analysis for the prediction of the myocardial viability.

**Dependent variable**	**Independent variable**	**B**	**β**	**Significance**	**Adjusted *R*^**2**^**
Viable myocardium (%)	Ischemic extent (%)	0.006	0.652	*P* <0.001	0.329
	Diastolic PSD (°)	−0.002	−0.315	*P* <0.01	
	NYHA	0.033	0.209	*P* <0.05	
Myocardial scar (%)	Ischemic extent (%)	0.004	0.392	*P* <0.001	0.373
	Diastolic PSD (°)	0.002	0.304	*P* <0.01	
	NYHA	−0.033	−0.202	*P* <0.05	

*NYHA, New York Heart Association functional classification; B, partial regression coefficient; β, standard regression coefficient*.

### Prognostic Value of LVMD

**Figure 5:**
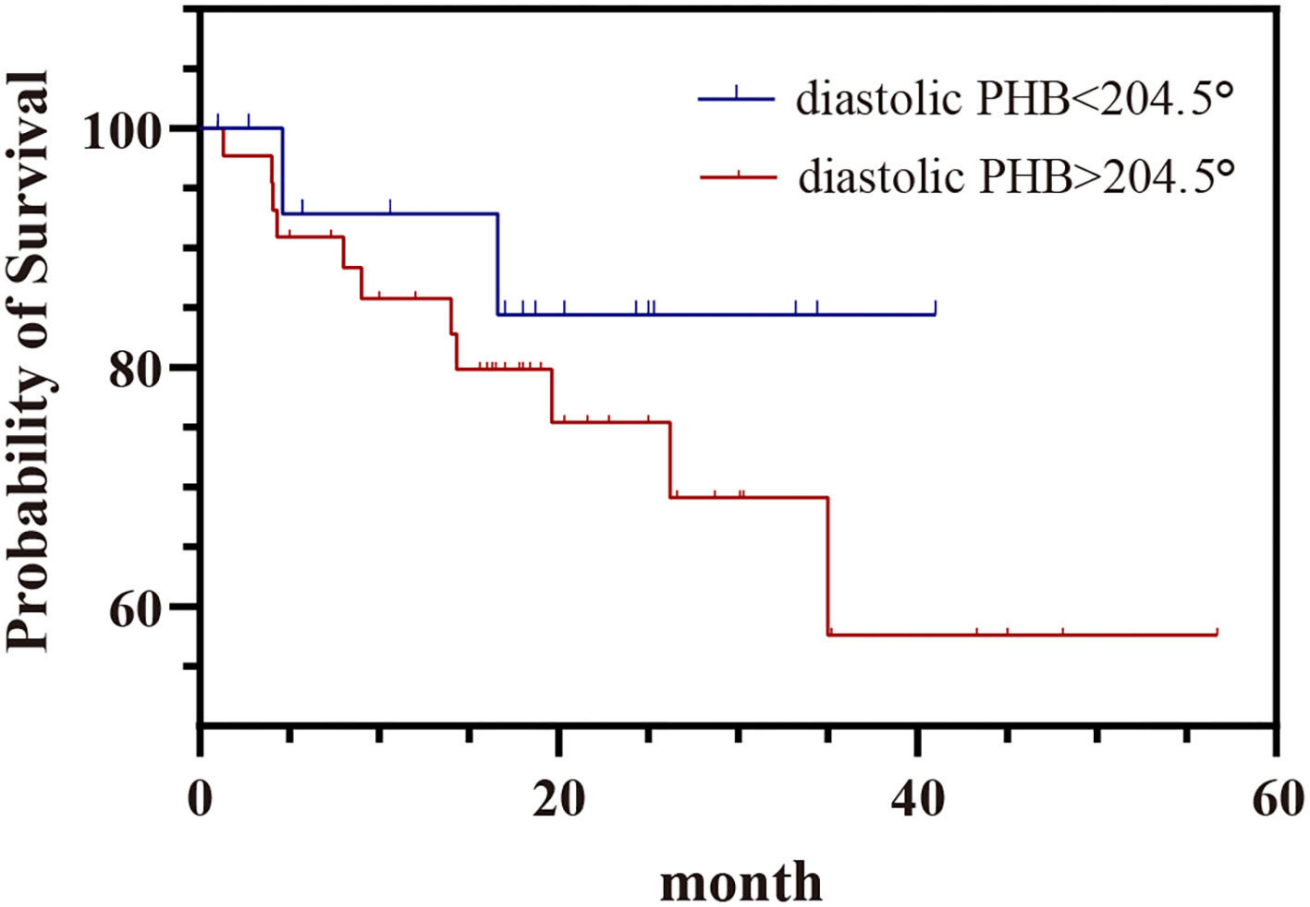
Kaplan–Meier survival curve of MACE in patients with diastolic PHB <204.5° and diastolic PHB > 204.5°. PHB, phase histogram bandwidth.

The mean follow-up time was 17.9 ± 12.9 months. Of the 79 patients, 65 had follow-up results, in which MACE occurred in 14 (17.7%), 44 did not have MACE, seven died of unknown causes. Kaplan-Meier survival curves of patients with diastolic PHB > 204.5° and PHB <204.5°were shown in Figure 5. The incidence of MACE in patients with diastolic PHB <204.5° (11.8%) was lower than that in patients with diastolic PHB > 204.5° (25.0%). However, there was no statistical difference in the incidence of MACE by log-rank test (*P* = 0.340).

## Discussion

This study focused on whether LVMD contributed to the diagnosis of viable myocardium and myocardial scar by Nitrogen-13 ammonia ECG-gated MPI. LVMD parameters differed among groups, which suggests the usefulness of LVMD to distinguish patients with different proportions of myocardial scar, which confirmed our hypothesis. Diastolic PHB contributed to predicting the presence of myocardial scar by the binary logistic regression model, and the cut-off value was 204.5°. Diastolic PSD, ischemic extent, and NYHA classification were identified in a multiple regression stepwise model to predict the degree of viable myocardium and myocardial scar. LVMD from phase analysis of Nitrogen-13 ammonia ECG-gated MPI was likely beneficial to the diagnosis of myocardial viability in CAD patients with LV systolic dysfunction. However, the value of LVMD as a predictor of MACE of CAD patients still needs further study.

The application of LVMD for CAD diagnosis was focused on the relationship between LVMD with coronary artery stenosis and myocardial perfusion defects in previous studies (9, 10, 24, 25). LVMD has been suggested to be associated with occult atherosclerosis in patients with normal coronary angiography but with reversible perfusion defects (9) and subclinical atherosclerosis (24). Moreover, the correlation between LVMD and myocardial perfusion defects assessed by SPECT overwhelmed the diagnosis of CAD by coronary angiography in patients with anginal chest pain and known or suspected CAD (10). Early detection and treatment of LVMD may slow the appearance of cardiac dysfunction in patients with ischemic heart disease (25). However, there is a paucity of studies linking myocardial viability and LVMD. It has been suggested that hibernating myocardium is an independent predictive factor of LVMD (15); thus, we hypothesized that LVMD may contribute to distinguishing the relative proportion of viable myocardium and myocardial scar by Nitrogen-13 ammonia gated MPI alone. Our results confirmed this hypothesis.

All the LVMD parameter values were larger in patients with a higher proportion of myocardial scar (Group 3) than in patients with a lower proportion (Group 2) and without myocardial scar (Group 1). A diastolic PHB cut-off of 204.5° predicted the presence of myocardial scar. The results suggested that combining LVMD with ischemic extent may provide clinicians with an intuitive impression on which patient has more proportion of viable myocardium. Previously, it was difficult to have a view of the degree of myocardial scar and viable myocardium on rest MPI alone. In the multiple stepwise regression analysis for the prediction of myocardial viability, we noticed the degree of myocardial scar increased and viable myocardium decreased with increasing diastolic PSD, indicating that diastolic PSD has diagnostic value in terms of myocardial viability in Nitrogen-13 ammonia ECG-gated MPI. There are several possible explanations. First, myocardial scar is one of the main features in the progression of CAD. It decreases regional myocardial electromechanical coupling efficiency and further reduces myocardial contraction or dilation coordination (26). Noise derived from scars in the raw data is significantly associated with PSD (27). Our results revealed that there was no statistical difference in LVMD parameters between Group 1 and Group 2, which may be due to limited number of patients in Group 1. The multiple stepwise linear regression model also indicated that ischemic extent, diastolic PSD, and NYHA functional classification should be taken into consideration together to predict viable myocardium and myocardial scar, rather than LVMD alone. In addition, the relatively low *R*^2^ values (0.329 and 0.373) of viable myocardium and myocardial scar indicated that ECG-gated MPI combined with phase analysis and NYHA classification were not completely equivalent to myocardial viability quantification assessed by myocardial perfusion/metabolism imaging.

Previous studies of phase analysis were focused on the application of systolic LVMD in SPECT; little attention was paid to either diastolic LVMD or the application of LVMD in PET, especially in Nitrogen-13 ammonia gated MPI. To date, the pathophysiology and clinical value of diastolic LVMD had not been studied extensively. Similar to systolic LVMD, diastolic LVMD could assess the phase and amplitude of regional myocardial relaxation by multiple Fourier transforms to quantify myocardial discoordination in diastole (23) and appears to provide incremental value in the prediction of adverse outcomes (28). In our study, both systolic and diastolic LVMD were included and it was suggested that diastolic LVMD was more strongly associated with myocardial viability, as seen in the diastolic phase histogram. Accordingly, the potential value of diastolic LVMD should be further investigated. Moreover, gPET has the advantage of higher spatial resolution to obtain a more accurate ischemic extent and has the potential to provide myocardial blood flow (MBF) (29). Previous studies suggested that the improvement of LVMD under stress was crucially associated with improved MBF homogeneity (30) and stress MBF was better correlated with LVMD than myocardial flow reserve (31). Thus, the relationship between LVMD and gPET quantitative cardiac functional parameters in Nitrogen-13 ammonia gated MPI is worth further study.

In a cohort of 489 patients with ischemic cardiomyopathy (14), worse LVMD in stress MPI as compared with rest was an independent predictor of all-cause mortality. Systolic and diastolic LVMD were associated with adverse outcomes in CAD patients when the cut-off value was determined by comparison with the normal population (28), and a significantly higher incidence of MACE was also found in patients with systolic LVMD (32). In the present study, although the incidence of MACE in patients with diastolic PHB >204.5° was 25.0%, higher than patients with diastolic PHB <204.5° (11.8%), the difference was not significant,which may owe to relatively small samples and short follow-up time. In addition, high proportion of patients with NYHA III and IV (51.9 and 29.1%, respectively) with poor prognosis may interfere the evaluation of the prognostic value of LVMD.

### Limitations

There were several limitations of our study. First, it is a retrospective study with limited number of patients. There were only seven patients in Group 1, which may influence detecting statistically significant differences in LVMD parameters between Group 1 and Group 2. There were reduced number of female patients, which may affect the evaluation of sex. Second, this study was a preliminary attempt to analyze the prognostic value of diastolic LVMD. Larger sample-sized study and longer follow-up time are needed to further confirm the prognostic value of diastolic LVMD.

## Conclusion

LVMD from Nitrogen-13 ammonia gated MPI had added diagnostic value for viable myocardium and myocardial scar. Increased LVMD did not reach a statistically significant predictive value. Further studies are needed.

## Data Availability Statement

The original contributions presented in the study are included in the article/supplementary material, further inquiries can be directed to the corresponding authors.

## Ethics Statement

The studies involving human participants were reviewed and approved by Human Ethics Committee of Union Hospital, Tongji Medical College, Huazhong University of Science and Technology. The patients/participants provided their written informed consent to participate in this study.

## Author Contributions

DZ conducted the analyses and was a major contributor in writing the manuscript. YL analyzed the images. LZ collected clinical information of all patients. FH and XT acquired GPET images. DJ contributed to revision. WZ provided suggestions about the idea. XL and CQ conceived the idea and contributed to analysis and revision. All authors read and approved the final manuscript.

## Funding

This work was supported by the National Natural Science Foundation of China (Nos. 81873906 and 81401444).

## Conflict of Interest

The authors declare that the research was conducted in the absence of any commercial or financial relationships that could be construed as a potential conflict of interest.

## Publisher's Note

All claims expressed in this article are solely those of the authors and do not necessarily represent those of their affiliated organizations, or those of the publisher, the editors and the reviewers. Any product that may be evaluated in this article, or claim that may be made by its manufacturer, is not guaranteed or endorsed by the publisher.
